# Characterization of total adenosine deaminase activity (ADA) and its isoenzymes in saliva and serum in health and inflammatory conditions in four different species: an analytical and clinical validation pilot study

**DOI:** 10.1186/s12917-020-02574-2

**Published:** 2020-10-12

**Authors:** María Dolores Contreras-Aguilar, Asta Tvarijonaviciute, Ingrida Monkeviciene, María Martín-Cuervo, Luis Guillermo González-Arostegui, Lorena Franco-Martínez, José Joaquín Cerón, Fernando Tecles, Damián Escribano

**Affiliations:** 1 Interdisciplinary Laboratory of Clinical Analysis (Interlab-UMU), Veterinary School, Campus of Excellence Mare Nostrum, University of Murcia. Campus de Espinardo s/n, 30100 Espinardo, Murcia, Spain; 2 Department of Anatomy and Physiology, Research Center of Digestive Physiology and Pathology, Veterinary Academy, Lithuanian University of Health Sciences, Tilzes str. 18, LT-47181 Kaunas, Lithuania; 3 Animal Medicine, Faculty of Veterinary Medicine of Cáceres, University of Extremadura, Av. De la Universidad s/n, 10005 Cáceres, Spain

**Keywords:** Adenosine deaminase, Isoenzymes, Saliva, Serum, Veterinary species

## Abstract

**Background:**

Measurement of adenosine deaminase (ADA) can provide information about cell-mediated immunity. This report’s objective was to study the enzymatic activity of total ADA (tADA) and its isoenzymes ADA1 and ADA2 in canine, equine, porcine, and bovine serum and saliva and their changes in different inflammatory situations in each species. Besides, an automated method for ADA2 measurement was developed and validated.

**Results:**

tADA was present in serum and saliva of healthy animals of the four species. Erythro-9-(2-hydroxy-3-nonyl) adenine (EHNA) concentration of 0.47 mM was needed for ADA1 inhibition in canine and porcine samples (serum and saliva) and bovine saliva, whereas for equine saliva 0.94 mM was needed. ADA2 activity was not detected in bovine serum and was very low or absent in equine serum and bovine saliva. An automated procedure to measure ADA2 consisting of adding EHNA to a commercial reagent for tADA measurement provided repetitive (coefficients of variation < 8.8% in serum and < 10% in saliva) and accurate (linearity of serial sample dilutions with *R*^2^ > 0.90) results, being equivalent to a manual incubation of the sample with EHNA at a similar concentration. Salivary tADA, as well as ADA1 and ADA2, were higher in dogs with leishmaniosis, horses with acute abdominal disease and pigs with lameness than in healthy animals. tADA and isoenzymes in saliva showed a positive significant correlation with serum ferritin in dogs (*r* = 0.602, *P* < 0.01; *r* = 0.555, *P* < 0.05; and *r* = 0.632, *P* < 0.01; respectively for tADA, ADA1 and ADA2) and serum C-reactive protein in pigs (*r* = 0.700, *P* < 0.01, for both tADA and ADA1; *r* = 0.770, *P* < 0.001, for ADA2), whereas salivary ADA2 significantly correlated with serum amyloid A in horses (*r* = 0.649, *P* < 0.01). In cows, salivary tADA and ADA1 significantly increased after calving, correlating with total white blood cell count (*r* = 0.487, *P* < 0.05, for both tADA and ADA1).

**Conclusions:**

The activity of total ADA and its different isoenzymes, can be measured in serum and saliva of dogs, horses, pigs and cows by a simple and fast procedure described in this report. When measured in saliva, these analytes correlated with other biomarkers of inflammation and it could potentially be used as a biomarkers of inflammation and immune activation in the species of this study.

## Background

Adenosine deaminase (ADA, EC number 3.5.4.4) is an ubiquitously expressed enzyme that can be found in several tissues and fluids and mediates the conversion of adenosine into inosine and of deoxyadenosine into deoxyinosine, playing a role in purine and pyrimidine metabolism [1]. ADA has two different isoenzymes. The isoenzyme ADA1 is mainly present in lymphoid tissue. It plays a role in the differentiation of B and T lymphocytes, as well as in maturation from monocyte to macrophage [2, 3], having an important role in adaptative immune function. ADA2 is the predominant in plasma in humans [4], but it has a lower affinity to adenosine compared with ADA1 [5–7]. Although poorly understood, ADA2 is probably involved in the haematopoietic system function [8, 9], being secreted by premonocytic cells as a growth factor for the monocyte lineage [5–7] and endothelial cells [9]. The involvement of both isoenzymes in the immune system is proven by the fact that their deficiency produces immune dysfunction. ADA1 deficiency leads to a severe combined immunodeficiency [10], and ADA2 deficiency can create several abnormalities, including vasculitis Behçet’s-like disease [11], immunodeficiency due to hypogammaglobulinemia, or cytopenias [12].

Total ADA (tADA) activity represents the sum of the two isoenzymes’ activities. It can be increased in serum in those diseases in which the number of T lymphocytes increases [13, 14]. Due to this fact, it has been used as a marker of cell-mediated immunity [15, 16] and chronic inflammation [17]. Several diseases have been reported to increase serum ADA activity in humans, including inflammation such as chronic tonsillitis, rhinosinusitis or otitis media [4], immune mediated disorders such as systemic lupus erythematosus [18, 19] or rheumatoid arthritis [20], and malignancies such as chronic lymphocytic leukemia [21], breast cancer [22] or bladder cancer [23]. However, the response of both isoenzymes could be different as observed in some diseases, such as rheumatoid arthritis, in which ADA2 increases, whereas ADA1 does not [20]. Serum tADA has also been measured in veterinary species showing increases in chronic inflammation in dogs [24] and cows [25]. In contrast, in a study measuring tADA in saliva and serum of pigs with lameness, a decreased tADA activity in serum was found [26].

Saliva is an organic fluid with potential usefulness for biomarkers determination, not only for oral diseases but also for systemic pathologies [27]. It can be easily and safely collected by untrained personnel, causing a minimum disturbance to the animal [28]. In humans, ADA activity was successfully measured in saliva being increased in local pathologies such as oral malignancies [29] or Sjögren’s syndrome [30], and systemic pathologies such as obesity [31]. In animals, increased tADA activity in saliva has been described in bitches with pyometra [32] and pigs with lameness [26], probably due to the presence of a systemic inflammatory process. Recently, tADA has been measured in saliva from sheep, cow, and horses [33–35].

In humans, the activity of ADA2 isoenzyme in serum is 100-fold lower than that of ADA1 [8], whereas it is undetectable in bovine serum [25, 36]. However, to the best of authors’ knowledge, ADA activity has not been characterized in the serum of other animal species. In addition, in saliva there are no studies about ADA isoenzyme activity characterization and distribution in any animal species except for the pig [37].

This report aimed to study the enzymatic activity of tADA, ADA1, and ADA2 in serum and saliva samples from four different animal species (dog, horse, pig, and cow). For the determination of the activity of ADA isoenzymes, an automated assay, in which the specific ADA1 inhibitor erythro-9-(2-hydroxy-3-nonyl) adenine (EHNA) was added to the reaction mixture in order to isolate ADA2 activity, was validated. In addition, changes on tADA, ADA1, and ADA2 were assessed in several inflammatory conditions. A correlation between these analytes and species-specific traditional inflammatory markers such as acute-phase proteins or total white blood cell count (WBC) was also performed.

## Results

### Values of tADA and its isoenzymes in serum and saliva in healthy individuals from different species

**Fig. 1:**
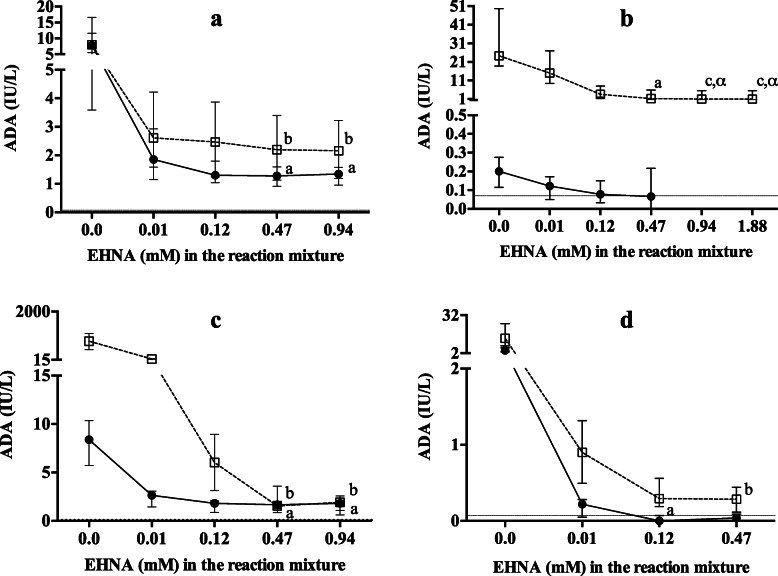
Changes in adenosine deaminase (ADA) activity after adding increasing concentrations of erythro-9-(2-hydroxy-3-nonyl) adenine (EHNA) in the different species of this study. Serum (solid circle) and saliva (empty square) from apparently healthy dogs (**a**, *n* = 5), horses (**b**, *n* = 5), pigs (**c**, *n* = 5) and cows (**d**, *n* = 5), with increasing EHNA concentration in the reaction mixture. The changes due to the presence of EHNA at different concentrations were assessed by Friedman’s, followed by Dunn’s multiple comparison tests. Letters indicate statistically significant results with the results obtained without EHNA (a: *P* < 0.05; b: *P* < 0.01; c: *P* < 0.001). Greek symbols indicate statistically significant results with the 0.01 mM EHNA concentration (α: *P* < 0.05). The horizontal dotted line shows the lower limit of quantification according to previously reported results [22, 28]. The best EHNA concentration for achieving complete ADA1 inhibition was selected based on that concentration able to reduce ADA activity and from which no greater significant inhibition was seen

Results obtained in dog samples are shown in Fig. 1a. Before incubation with EHNA, serum and saliva samples showed similar ADA activities. In both serum and saliva samples, a significant inhibition was achieved with 0.12 mM EHNA in the reaction mixture, but complete inhibition was achieved with 0.47 mM in both saliva and serum. When 0.47 mM EHNA was used, the median (25th–75th percentiles) for ADA1 and ADA2 in serum was 6.32 (5.66–9.91) IU/L and 1.28 (1.21–1.50) IU/L, respectively. In saliva, those values were 5.77 (3.76–6.58) IU/L and 2.20 (1.50–3.24) IU/L for ADA1 and ADA2, respectively.

Results obtained in horse samples are shown in Fig. 1b. In serum, tADA activity was very low before adding EHNA, with 1/5 samples having values under the LLOD. Since no change was observed between 0.12 and 0.47 mM EHNA, and most values were under the LLOD of the method, a higher EHNA concentration was not tested. In contrast, saliva samples showed higher ADA activities than the LLOD of the method in all the samples. The statistical analysis showed that ADA was significantly inhibited with 0.47 mM EHNA, although a concentration of 0.94 mM would be most appropriate for complete ADA1 inhibition. At 0.47 mM EHNA, the median (25th–75th percentiles) for ADA1 and ADA2 in serum was 0.12 (0.06–0.14) IU/L and 0.07 (0.00–0.21) IU/L respectively. In saliva, those values at 0.94 mM EHNA concentration were 18.70 (18.29–39.34) IU/L and 0.98 (0.61–5.47) IU/L for ADA1 and ADA2, respectively.

Results obtained with pig samples are shown in Fig. 1c. Saliva had a much higher ADA activity than serum. In serum, significant inhibition was observed with 0.47 mM EHNA, with no further reduction in activity with a higher concentration. Similar results were found in saliva. All results were above the LLOD of the method. At 0.47 mM EHNA, median (25th–75th percentiles) for ADA1 and ADA2 in serum were 7.67 (4.74–8.30) IU/L and 1.67 (1.00–1.91) IU/L, respectively. In saliva, those values were 782.20 (496.65–1078.27) IU/L and 1.53 (1.30–2.35) IU/L for ADA1 and ADA2, respectively.

Results obtained in cow samples are shown in Fig. 1d. Before incubation with EHNA, serum, and saliva samples provided similar tADA results. In serum, inhibition was considered significant compared to initial values at 0.12 mM EHNA, with 4/5 values under the LLOD; no more changes were observed with a higher concentration. In saliva, 0.47 mM EHNA was required to achieve a significant inhibition, with no samples below the LLOD. Since no changes were observed between 0.12 and 0.47 mM EHNA in both serum, and saliva samples, a higher EHNA concentration was not tested. At 0.47 mM EHNA, the median (25th–75th percentiles) for ADA1 in serum was 4.20 (3.87–7.56) IU/L, whereas ADA2 was negligible. In saliva, the values were 13.55 (6.13–20.96) IU/L for ADA1, and 0.29 (0.13–0.44) IU/L for ADA2.

### Validation of the automated assay for ADA2 measurement

The intra-assay CV was lower than 8.8% in serum and 10% in saliva (Table 1). Linearity under dilution approach showed *R*^2^ > 0.90 in all cases (Table 2). LLOD was set at 0.07 UI/L. The automated ADA2 determination was not validated in serum from horses and cows since values were under the LLOD of the assays.

**Table 1:** Intra-assay precision results obtained from two pools of serum and saliva with high and low ADA2 activity from different species. They were obtained from two pools of serum from dogs and pigs and two pools of saliva from dogs, horses, pigs, and cows with high and low adenosine deaminase 2 activity. Mean ± standard deviation (coefficient of variation) from 5 replicates of each pool of samples and species are indicated. Mean and standard deviations are expressed in IU/L, coefficients of variation in %

	Serum	Saliva
Dog		
High	1.79 ± 0.10 (5.63)	3.83 ± 0.13 (3.43)
Low	0.62 ± 0.03 (4.60)	0.87 ± 0.09 (9.96)
Horse		
High	–	4.77 ± 0.14 (2.89)
Low	–	0.66 ± 0.06 (8.60)
Pig		
High	4.30 ± 0.18 (4.15)	6.06 ± 0.06 (0.97)
Low	0.91 ± 0.08 (8.72)	0.67 ± 0.04 (5.41)
Cow		
High	–	1.99 ± 0.11 (5.75)
Low	–	0.24 ± 0.02 (6.59)

**Table 2:** Linearity under dilution for the automated measurement of adenosine deaminase isoenzyme 2 (ADA2). Results were obtained from two pools of serum from dogs and pigs and two pools of saliva from dogs, horses, pigs and cows with high and low ADA2 activity

Species	Sample	ADA2 (IU/L)	Slope	Y-intercept	***R***^**2**^	***P***
Dog	Saliva	3.90	1.14 ± 0.07	−0.04 ± 0.08	0.98	< 0.001
		0.64	1.35 ± 0.15	−0.02 ± 0.03	0.95	< 0.001
	Serum	1.79	1.14 ± 0.10	−0.01 ± 0.05	0.95	< 0.001
		0.98	1.16 ± 0.14	−0.01 ± 0.05	0.94	< 0.01
Horse	Saliva	4.77	1.00 ± 0.12	−0.15 ± ± 0.17	0.91	< 0.001
		0.66	1.02 ± 0.05	0.05 ± 0.04	0.99	< 0.001
Pig	Saliva	6.06	1.02 ± 0.03	0.00 ± 0.05	0.99	< 0.001
		0.67	1.04 ± 0.13	−0.04 ± 0.04	0.92	< 0.001
	Serum	4.30	1.38 ± 0.06	−0.35 ± 0.07	0.99	< 0.001
		0.91	1.34 ± 0.13	−0.21 ± 0.06	0.95	< 0.001
Cow	Saliva	2.20	1.65 ± 0.14	0.06 ± 0.09	0.96	< 0.001
		0.56	1.41 ± 0.21	−0.05 ± 0.04	0.92	< 0.01

*R*^*2*^: Coefficient of linear regression; *P*: *P* value

**Fig. 2:**
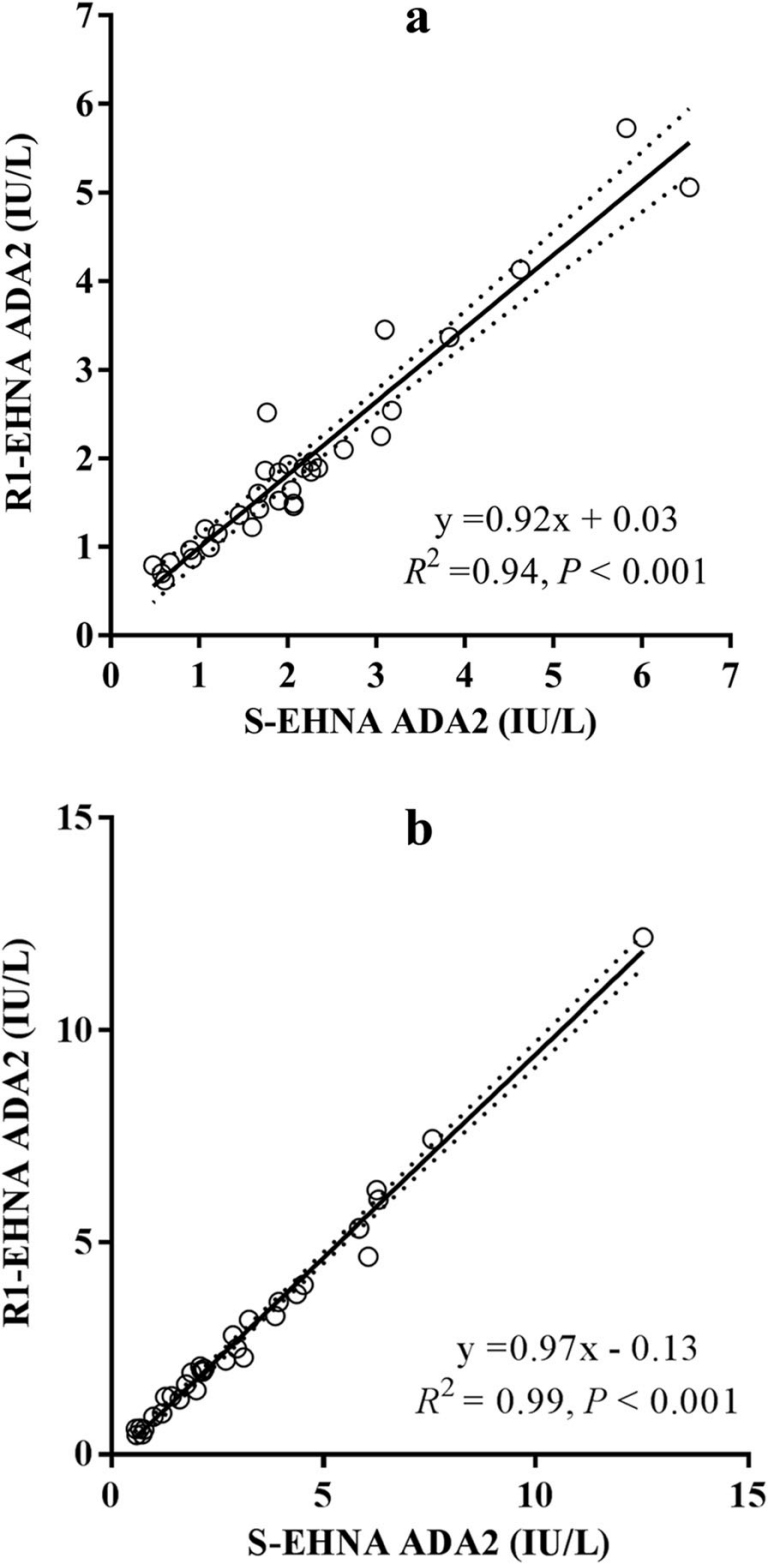
Linear regression between adenosine deaminase 2 (ADA2) results with manual vs. automated inhibition. Empty circles show pair of results obtained in serum (**a**) and saliva (**b**) when EHNA was added to samples (S-EHNA) vs. when EHNA was added to the commercial reagent 1 (R1-EHNA) to achieve a final concentration of 0.47 mM. Samples from 33 pigs (15 apparently healthy and 18 with disease) were used. The continuous line shows linear regression, and the dotted lines show the 95% confidence interval. *R*^*2*^: Coefficient of linear regression

**Fig. 3:**
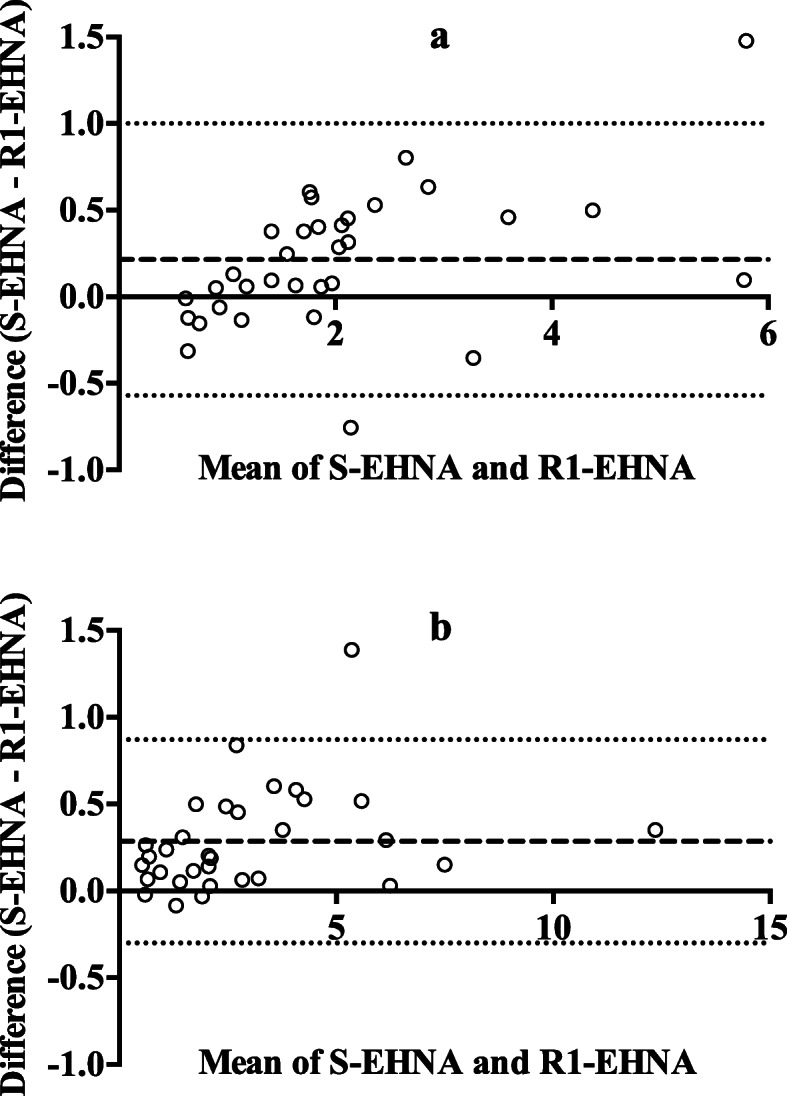
Bland-Altman plot between adenosine deaminase 2 (ADA2) results with manual vs. automated inhibition. Empty circles show pair of results obtained in serum (**a**) and saliva (**b**) when EHNA was added to samples (S-EHNA) vs. when EHNA was added to the commercial reagent 1 (R1-EHNA) to achieve a final concentration of 0.47 mM. Samples from 33 pigs (15 apparently healthy and 18 with disease) were used. Separated lines show bias and dotted lines the 95% limits of agreement

For the comparison between manual and automated ADA2 determination protocols in porcine samples, a final EHNA concentration in the reaction mixture of 0.47 mM was selected since the previous results showed that this concentration was able to inhibit ADA1 isoenzyme in both serum and saliva samples completely. Linear regression between ADA2 results obtained with the manual incubation and the automated procedure showed slope significantly close to 1 and Y-intercept substantially close to zero, with an *R*^2^ > 0.94 (*P* < 0.001) for serum and *R*^2^ > 0.99 (*P* < 0.001) for saliva (Fig. 2). Bland-Altman plots showed a bias of 0.21 ± 0.40 IU/L for serum and 0.29 ± 0.30 IU/L for saliva (Fig. 3).

### Clinical validation

Results of the different trials performed to evaluate changes in ADA in inflammatory conditions are shown in Table 3, and correlation coefficients between serum and salivary tADA, ADA1 and ADA2 with the different inflammatory biomarkers are shown in Table 4. No statistically significant differences were observed between healthy dogs and dogs with leishmaniasis in serum tADA or its isoenzymes. However, salivary tADA, as well as both isoenzymes ADA1, and ADA2, were statistically higher in the dogs with leishmaniasis, showing moderate correlation with serum ferritin (*r* = 0.60, *P* < 0.01, for tADA; *r* = 0.56, *P* < 0.05, for ADA1; and *r* = 0.63, *P* < 0.01, for ADA2).

**Table 3:** Adenosine deaminase activity in animals with and without inflammatory conditions. Total adenosine deaminase (tADA), and isoenzymes ADA1 and ADA2, obtained in healthy animals and dogs with clinical leishmaniosis, horses with the acute abdominal disease, pigs with lameness and healthy cows before and after calving. Inflammation was assessed by the measurement of ferritin in dogs, serum amyloid A (SAA) in horses, C-reactive protein (CRP) in pigs, and haptoglobin (Hp) and white blood cell count (WBC) in cows. All results are expressed in the median (25th–75th percentiles). Units: IU/L (ADA), μg/L (ferritin), μg/mL (SAA and CRP), mg/L (Hp) and 10^9^/L (WBC). Unpaired Mann-Whitney test was used to compare tADA and isoenzymes results between healthy and diseased animals. The Wilcoxon signed-rank test was used to compare tADA and isoenzymes results between the two different measurements performed in cows

	Serum		Saliva	
Dogs	Healthy	Canine leishmaniosis	Healthy	Canine leishmaniosis
	*N* = 10	*N* = 10	*N* = 10	*N* = 10
Ferritin	123.20 (106.60–145.00)	707.20*** (299.80–1483.00)		
tADA	12.90 (8.60–16.83)	9.05 (7.85–16.25)	4.25 (2.75–5.65)	10.60* (3.55–12.18)
ADA1	12.12 (7.77–15.60)	8.78 (7.24–14.70)	2.81 (1.78–4.54)	7.49* (2.07–10.15)
ADA2	1.00 (0.69–1.25)	0.61 (0.42–1.19)	1.16 (0.87–1.61)	2.11* (1.08–3.00)
Horses	Healthy	Acute abdominal disease	Healthy	Acute abdominal disease
	*N* = 10	*N* = 10	*N* = 10	*N* = 10
SAA	1.00 (1.00–1.00)	129.60*** (124.10–230.80)		
tADA	0.23 (0.16–0.35)	0.43 (0.01–0.90)	46.30 (18.74–61.48)	162.20* (47.00–379.30)
ADA1	0.15 (0.05–0.20)	0.28 (0.01–0.52)	43.84 (18.26–60.80)	158.60* (42.36–368.80)
ADA2	0.06^a^ (0.00–0.19)	0.01^a^ (0.00–0.51)	0.90 (0.26–2.50)	5.35** (2.02–10.53)
Pigs	Healthy	Lameness	Healthy	Lameness
	*N* = 10	*N* = 10	*N* = 10	*N* = 10
CRP	6.60 (3.73–13.78)	72.50*** (41.03–101.00)		
tADA	10.42 (8.34–13.70)	9.61 (6.86–10.47)	495.10 (347.00–823.40)	1829.00** (814.50–2683.00)
ADA1	8.87 (7.11–12.53)	6.29* (4.07–7.91)	493.00 (346.40–822.40)	1823.00** (811.60–2677.00)
ADA2	1.40 (0.79–1.91)	2.31* (1.45–4.36)	1.12 (0.55–2.06)	4.33*** (2.36–8.61)
Cows	Before calving	At calving	Before calving	At calving
	*N* = 10	*N* = 10	*N* = 10	*N* = 10
WBC	6.46 (5.76–7.55)	10.41** (9.03–13.58)		
Hp	0.00 (0.00–5.85)	4.82* (0.00–31.60)		
tADA	7.14 (6.01–8.21)	8.79* (6.93–13.47)	5.51 (2.93–6.96)	22.40** (13.19–28.61)
ADA1	7.14 (6.01–8.21)	8.79* (6.93–13.47)	5.20 (2.55–6.57)	22.30** (12.63–28.40)
ADA2	0.00^a^ (0.00–0.00)	0.00^a^ (0.00–0.00)	0.36 (0.13–0.46)	0.14 (0.02–0.29)

^a^under the lower limit of detection of the assay; *: *P* < 0.05; **: *P* < 0.01; ***: *P* < 0.001

**Table 4:** Correlations between total adenosine deaminase (tADA), and isoenzymes ADA1 and ADA2, with the inflammatory biomarker used in each species. Spearman correlation coefficients (*r*) are expressed

	Ferritin (dog)	SAA (horse)	CRP (pig)	WBC (cow)	Hp (cow)
**Serum**					
**tADA**	−0.06	0.10	−0.36	0.42	−0.14
**ADA1**	−0.01	0.20	−0.51*	0.42	−0.14
**ADA2**	−0.19	−0.11	0.33	0.06	−0.06
**Saliva**					
**tADA**	0.60**	0.43	0.70**	0.49*	0.23
**ADA1**	0.56*	0.43	0.70**	0.49*	0.27
**ADA2**	0.63**	0.65**	0.77***	−0.22	−0.03

*SAA* Serum amyloid A, *CRP* C-reactive protein, *WBC* White blood cell count, *Hp* Haptoglobin. Statistical analysis: * = *P* < 0.05; ** = *P* < 0.01; *** = *P* < 0.001

In horses, serum provided tADA activity values under the LLOD in 2/10 healthy animals and 3/10 with colic, with no statistical differences between groups. ADA2 in serum was under the LLOD in all samples. In contrast, all values found in saliva were over the calculated LLOD of the method, with values of tADA and both isoenzymes being statistically higher in horses with colic. ADA2 in saliva moderately correlated with serum SAA (*r* = 0.65, *P* < 0.01).

In pigs, serum ADA1 was statistically lower in the diseased group than in the healthy group, whereas serum ADA2 was statistically higher. ADA1 in serum showed a moderate negative correlation with serum CRP (*r* = − 0.51, *P* < 0.05). In saliva, tADA and both isoenzymes were statistically higher in the diseased group. Salivary tADA, ADA1 and ADA2 showed a significant high correlation with serum CRP (*r* = 0.70, *P* < 0.01, for both tADA and ADA1; *r* = 0.77, *P* < 0.001, for ADA2).

In cows, tADA in serum showed a significant increase at calving, being entirely due to ADA1, since ADA2 isoenzyme provided negligible results in all cases. In saliva, tADA activity showed a 4-fold increase at calving, as well as ADA1 isoenzyme. No significant changes were observed in the isoenzyme ADA2. Salivary tADA and ADA1 showed a low positive correlation with WBC count (*r* = 0.49, *P* < 0.05), whereas no correlation was observed with Hp.

## Discussion

EHNA is a specific ADA1 isoenzyme inhibitor that, at a proper concentration, can completely inhibit ADA1 isoenzyme whereas ADA2 remains unaffected [38]. In our report, the EHNA concentration was selected for each species as the minimum concentration able to inhibit ADA1 activity totally. The results indicated that an EHNA concentration of 0.12 mM in the reaction mixture would be enough to completely inhibit ADA1 isoenzyme in cow serum. However, in canine and porcine samples (both serum and saliva), as well as in bovine saliva, a higher concentration of at least 0.47 mM in the reaction mixture would be needed. A concentration of 0.94 mM in the reaction mixture would be more appropriate in equine saliva, since it produced an additional significant inhibition than 0.47 mM. In this species, no additional reduction on activity was observed with a concentration of 1.88 mM in the reaction mixture. These concentrations are higher than those recommended in the bibliography for humans of 0.1 mM in the reaction mixture [38]. Altuğ et al. [25] used an EHNA concentration of 0.2 mM in the reaction mixture for measuring ADA2 in serum from cows, which is also higher than the concentration recommended for humans. However, our results would indicate that 0.12 mM would be enough in this serum of cows. Therefore, the sensitivity to the inhibitor could depend on the animal species and the sample, being bovine serum the most sensitive and equine saliva the less. To the authors’ knowledge, no studies did assess the molecular structure of ADA proteins in different species. This could contribute to explain the different sensitivity to the ADA inhibitor (EHNA) observed in different sample types and between species. 

An automated procedure of quantification of ADA2 was developed in our study. For this purpose, EHNA was added directly to a commercially available reagent designed to measure tADA, to achieve a final concentration in the reaction mixture that was adequate for the species to be tested. This assay gave adequate values for imprecision and accuracy in all the species tested. It could be used together with the previously validated tADA assay for the measurement of the different isoenzymes in the species of our study. For this purpose, a three-step procedure is proposed: (1) the sample is measured for evaluation of tADA with the commercial reagent by a previously validated assay [26, 32, 34]; (2) the sample is measured again with the commercial reagent adding EHNA at the appropriate concentration for ADA2 estimation; and (3) the isoenzyme ADA1 is calculated by the difference between measurements of step 1 and 2. This procedure showed a low bias compared to the manual inhibition of samples. Therefore it would have no implication in clinical decision making; also it has the advantage that it can be fully automated, would avoid pipetting errors, and allows achieving results faster than with a manual inhibition of the samples.

Once isoenzymes distribution was studied in saliva and serum of the different animal species, their use as inflammatory biomarkers was assessed. Reliable inflammatory biomarkers are available for each species studied in this research, such as acute phase proteins. However, ADA, due to its lymphoid origin, is considered a marker of cell-mediated immunity [15–17], whereas acute phase proteins are more related to innate immunity [39]. Therefore, ADA could potentially provide additional information to that provided by the acute phase proteins. In addition, currently, the method for the measurement of ADA is cheaper than those used for the measurement of acute phase proteins, especially in saliva where the use of more sensitive assays such as time resolved fluorometry is needed because of their very low concentration [40].

In dogs, our results indicated that both canine serum and saliva had a similar tADA activity, being ADA1 the main contributor. When our assay was applied in dogs with leishmaniosis, no changes were detected in serum ADA with respect to healthy individuals. In contrast, tADA, as well as its isoenzymes, were higher in the saliva of dogs with canine leishmaniosis, correlating significantly with serum ferritin. This fact would indicate that ADA in saliva can increase in inflammatory conditions in dogs, in line with a previous report made in bitches with pyometra, which presented higher salivary tADA values than healthy dogs [32].

In our report, tADA value in horse serum was low but over the LLOD of the assay, or absent. The lack of ADA activity in the serum of horses has been previously reported [41]. Over a 14-fold lower ADA activity has been found in horse lymphocytes when compared with humans [42], which could influence the very low ADA activity in horse serum. In contrast, horse saliva provided an abundant ADA activity, being ADA1 the main contributor. Further studies should be performed to find the source of this activity in saliva, taking into account the low ADA activity in serum and lymphocytes in this species. Higher values of salivary tADA and isoenzymes were detected in horses with acute abdominal disease compared to healthy ones, especially in the case of ADA2. In fact, ADA2 showed a significant positive correlation with SAA in contrast to tADA and ADA1, and it could be considered as an inflammatory biomarker in horses. Therefore, although ADA1 is usually responsible for main changes in tADA activity, in some specific situations, such as in horses with acute abdominal disease, the measurement of ADA2 could be of interest and provide additional information, as reported in humans [20].

Pigs showed the highest ADA values, especially in saliva, where tADA was over 100-fold higher than in serum, in agreement with previous reports [26]. The reason for this high ADA activity in porcine saliva is unknown and should be further studied. In our report, ADA1 was the main contributor to the tADA activity in both serum and saliva. It was in agreement with a recent report [33] but in contrast with a previous one that found ADA2 as the predominant form in porcine saliva [43]. In saliva, significant increases were found in tADA and ADA1 in pigs with lameness compared with healthy pigs, but these were not detected in serum. In serum, only significant increases of ADA2 were found that were of lower magnitude than those found in saliva. Therefore, saliva would a better sample than serum for detecting increases of tADA and isoenzymes in pigs with lameness. The high correlation observed between tADA, its isoenzymes and serum CRP, suggests that salivary ADA activities could be used as inflammatory biomarkers in this species.

In cows, we did not find ADA2 activity in serum, being these results in line with other reports [25, 36]. In saliva, tADA activity was of similar magnitude as in serum, and saliva from some healthy individuals did present ADA2 activity, although in low amounts. In our study, calving significantly increased ADA1 isoenzyme in both serum and saliva. An increase of ADA in serum after calving had been previously described [4], and therefore ADA has been proposed to be a marker of a proinflammatory status of the animal due to calving [44]. To the authors’ knowledge, this is the first report in which salivary ADA has also been reported to increase after calving in cows, being this change of higher magnitude of that reported in serum. The correlation observed between salivary ADA, and the WBC count would indicate that also salivary ADA could be used as a biomarker of inflammation in this species. The lack of correlation with serum Hp could be due to the slow response of this acute-phase protein. The measurement of inflammatory biomarkers such as Hp or SAA, can help detect inflammatory conditions such as metritis or mastitis at the peripartum period [45]. Further studies should be made to evaluate whether ADA could detect similar pathological conditions in this species.

The primary limitations of this study were the relatively low number of animals used for the clinical validation and that only one inflammatory condition was studied in each species. Therefore, it should be considered as a pilot one, and further studies involving a large number of animals would be desirable in the future. In addition, different diseases or inflammatory conditions should also be studied, including those appearing during calving. This would allow a better knowledge about how tADA and its isoenzymes behave, as well as their possible use as biomarkers of disease.

## Conclusions

Total ADA activity, and its different isoenzymes, could be measured in both serum and saliva in dogs, horses, pigs, and cows by a simple and fast automated procedure described in this report. It should be taken into account that in this procedure, the concentration EHNA, which is used to inhibit ADA1, should be appropriately adjusted to the animal species in which it is going to be applied. Total ADA activity (and consequently its isoenzymes) is higher in saliva than in the serum of healthy horses and pigs but has similar values in saliva and serum in dogs and cows. Saliva seems a promising organic fluid that can be used to measure ADA activity. It can be easily and safely collected, even by untrained personnel, causing a minimum disturbance to the animal and ADA values correlates with other gold standard inflammatory biomarkers in different species. Further studies involving more animals and pathological conditions should be performed to assess the use of tADA and its isoenzymes as biomarkers of disease.

## Methods

### Sampling

In all cases, saliva was obtained prior to blood to avoid any possible influence of stress associated with blood collection on the saliva results. Saliva was collected using Salivette tubes (﻿Sarstedt, Aktiengesellschaft & Co. D-51588 Nümbrecht, Germany) containing a sponge (﻿Esponja Marina, La Griega E. Koronis, Madrid, Spain) instead of a cotton swab. The animals were allowed to chew the sponge until thoroughly moist with the help of a flexible thin metal rod. Then, the sponge was placed into the Salivette tube. Venous blood was obtained from venipuncture of the jugular (dogs, horses, and pigs) or caudal (cows) veins, using tubes without additive (BD Vacutainer, Franklin Lakes, NJ, USA) and allowed to clot. All samples were kept in ice until arrival at the laboratory for processing (less than 2 h).

Once at the laboratory, all saliva samples were visually checked, and those suspected of blood contamination (reddish coloration) were excluded. The saliva samples were centrifuged (Universal 320R, Hettichzentrifugen, Tuttlingen, Germany) at 3000 x g and 4 °C for 10 min. The supernatant was collected in Eppendorf tubes of 1.5 mL, and the sediment discarded. Blood tubes were centrifuged similarly to those of saliva, and serum was aliquoted in Eppendorf. Saliva and serum specimens were stored at − 80 °C until analysis.

### ADA assay

ADA was analyzed with a commercially available spectrophotometric automated assay (Adenosine Deaminase assay kit, Diazyme Laboratories, Poway, CA, USA). The methodology’s principle can be summarized as follows: 1) Reagent 1 containing 4-aminoantipyrine (4-AA), purine nucleoside phosphorylase (PNP), xanthine oxidase (XOD) and peroxidase in Tris-HCl buffer (pH 8.0) is pipetted to the reaction cuvette with the simple. 2) Then, the Reagent 2 containing the substrate adenosine and N-ethyl-N-(2-hydroxy-3-sulfopropyl)-3-methylaniline (EHSTP) in Tris-HCl buffer (pH 4.0) is added. The substrate adenosine is deaminated to inosine by ADA present in the sample. PNP then converts inosine to hypoxanthine, which is then transformed into uric acid and hydrogen peroxide (H2O2) by XOD. The amount of H_2_O_2_ produced in the reaction is proportional to the ADA activity in the sample. It is quantified by reaction with EHSTP and 4-AA in the presence of peroxidase, leading to a quinine dye kinetically monitored at a 550 nm wavelength [46]. This method was adapted to an automated analyzer (Olympus AU400, Olympus Diagnostica GmbH, Ennis, Ireland) following the manufacturer’s protocol. Given that, in some species, salivary ADA activity is low, sample volume was increased to allow its determinations. The lower limit of detection (LLOD) of this method, established in a previous study, was 0.07 IU/L [32].

For isoenzyme determinations, the specific ADA1 inhibitor EHNA (Merck KGaA, Darmstadt, Germany) was used. At a proper concentration, EHNA inhibits ADA1 isoenzyme, whereas ADA2 remains unaffected [38]. Therefore, tADA and ADA2 isoenzyme can be determined when samples are analyzed in the absence and presence of EHNA, respectively. The isoenzyme ADA1 is calculated from the difference between both measurements.

### Optimization of EHNA concentration for ADA2 measurement in serum and saliva in different species

To determine the appropriate concentration of EHNA that should be used in each species for total inhibition of ADA1 isoenzyme, the following samples were used:

#### Dogs

Samples were obtained from five healthy Beagle dogs (*Canis lupus familiaris*). All dogs were neutered males, 3.5 ± 0.8 years old, and 26.0 ± 7.1 Kg body weight. The animals were located in the Experimental Farm of the University of Murcia (Murcia, Spain). Animals did not show any clinical signs after physical examination and had within reference intervals values on routine haematological or biochemistry analyses. Serum C-reactive protein (CRP) concentration was measured as previously described [47]. All animals had CR*P* values < 10 μg/mL, indicating the absence of systemic inflammation as previously reported [48].

#### Horses

Samples were obtained from five healthy horses (*Equus caballus*), one stallion and four ﻿geldings, a mean age 10.0 ± 5.1 years old, with body condition score (BCS) 3.4 ± 0.5, including three Spanish horses, one Spanish Arabian and one Warmblood. Horses showed no clinical signs of pain or discomfort after a physical examination and did not show any haematological or biochemistry alteration. The serum levels of the acute phase protein serum amyloid A (SAA) were measured as described [49] and used as marker of acute systemic inflammation. SAA concentration was < 2.3 μg/mL in all animals.

#### Pigs

Samples were collected from five apparently healthy growing pigs (*Sus scrofa domesticus*), Large White x Large White males with 2–3 months-old in the last phase of fattening, housed in the Experimental Farm of the University of Murcia (Murcia, Spain). Animals did not show any clinical signs after physical examination and had within reference intervals values on routine haematological or biochemistry analyses. Serum CRP concentration, measured as previously described [37], was used as a marker of systemic inflammation. All animals have CRP values < 20 μg/mL.

#### Cows

Samples were obtained from five Holstein dairy cows (*Bos taurus*), lactation 3.5 ± 1.0, mean age 5.3 ± 1.4 years old, days in milk 234.8 ± 9.4, from a commercial dairy herd located in the southeast of Spain. The animals were healthy at physical examination and did not show any haematological or biochemistry alteration. The acute phase protein haptoglobin (Hp) measured as previously described [35] was used as an indicator of inflammation, giving all animals values < 20 mg/L [50].

Each saliva and serum sample was separated into five aliquots. Then, EHNA was added to the saliva and serum samples at increasing concentrations, whereas an equal volume of diluent was added to one aliquot that was used as control. The concentration of EHNA by which ADA1 activity was wholly inhibited varied between the studied species and was determined on both 1) the concentration was able to decrease tADA activity, and 2) a no further significant reduction in the enzymatic activity was obtained with a higher concentration.

### Development and validation of an automated assay for ADA2 isoenzyme measurement

An automated assay for the measurement of the ADA2 isoenzyme was developed: the adequate inhibitory concentration of EHNA previously calculated for each species was added to the reagent 1. In each species, a similar volume of samples obtained from 10 different animals (five with low and five with high ADA2 activity) were mixed to prepare two pools of serum and two pools of saliva with different ADA2 activity. Intra-assay imprecision and linearity under dilution were evaluated in serum and saliva samples from the various species by calculating the intra-assay coefficient of variation (CV) and linear regression coefficient, following previously published protocols [26, 32].

### Comparison between the automated and manual measurement of ADA2 activity

The results obtained with the automated procedure were compared with those obtained after the manual addition of the inhibitor. For this approach, the pig was selected as a model because of its high activity in saliva samples. For this purpose, serum and saliva samples with low (*N* = 15) and high (*N* = 18) ADA2 activity were selected. ADA2 isoenzyme was analyzed manually adding EHNA to the samples and automatically by adding EHNA to the reagent 1. Such concentrations in both procedures the same final concentration of EHNA in the reaction mixture was achieved.

### Clinical validation

For this purpose, tADA and its isoenzymes were measured using the fully automated method in the following samples:

#### Dog

20 samples were included and divided into two groups: 1) 10 samples were collected from healthy client-owned dogs belonging to the staff of the Animal Medicine and Surgery Department of the University of Murcia. They were 3.9 ± 1.5 years old, with BCS 4.0 ± 1.0, and included three Retrievers, three mixed-breed dogs, and one of the following breeds: Beagle, French bulldog, Scottish terrier, and Brie shepherd. All were neutered males apparently healthy after physical and haematological examinations. 2) 10 samples were collected from client-owned dogs admitted to the Veterinary Teaching Hospital of the University of Murcia and diagnosed with naturally occurred *Leishmania infantum* infection based on clinical signs and test results. The group with leishmaniosis included three mixed breed dogs and one of the following breeds: Retriever, French bulldog, Collie, Beagle, Irish setter, German shepherd and Rottweiler. There were five males and five females, with 3.0 ± 1.0 years-old, and BCS 2.7 ± 0.5. The clinical signs described in the 10 dogs with leishmaniosis included lymphadenopathy, and anaemia (1/10), skin lesions and uveitis (2/10), weight loss and hypoalbuminaemia (3/10), and hyperglobulinaemia (6/10). The diagnoses were based on positive polymerase chain reaction (PCR) and serology results. The concentrations of the acute phase protein ferritin in serum, a biomarker of systemic inflammation in this disease, were analyzed in healthy and diseased animals as previously described [51]. All healthy individuals had values < 190 μg/L.

#### Horse

Samples from 20 horses (10 considered as healthy after physical and blood examinations, and 10 with acute abdominal pain) were included. The healthy animals were male horses admitted for castration or routine health check. They included different breeds (seven Spanish horses, one Warmblood, and two crossbreed), mean age 8.0 ± 4.2 years-old, and BCS 3.5 ± 0.4. They showed no clinical signs of abdominal pain or other diseases during physical examination, as well as haematological or biochemical abnormalities. The group of diseased animals consisted of horses with acute abdominal disease. This group included animals with different breeds (five Spanish horses, two Warmblood horses, one Lusitanian horse, one Holsteiner, and one crossbreed) all males, mean age 11.3 ± 3.3 years-old, and BCS 3.4 ± 0.7. The diagnoses were based on clinical history, physical examination, haematology and plasma biochemistry, transabdominal ultrasonography, rectal examination, nasogastric intubation, and laparotomy findings in surgical cases. The following diagnoses were obtained: three colon impaction with large colon displacement, three stomach impaction, one nephrosplenic entrapment, one impaction of the pelvic flexure, one large colon displacement, and one enteritis. SAA was measured as a marker of acute systemic inflammation in all animals. All healthy animals showed SAA values < 2.3 μg/mL.

#### Pig

Samples from 20 animals (Large White x Large White males with 2–3 months-old in the last phase of fattening) housed in the Experimental Farm of the University of Murcia (Spain) were used. The healthy group was composed of 10 apparently healthy pigs after physical examination at the farm. The diseased group was formed of 10 lame pigs. The presence of lameness was considered based on the observation of the animals according to the scoring system published by Main et al. [52]. An animal was considered lame when a lameness score of ≥1 was achieved. Serum CRP concentration was used in all animals as a marker of systemic inflammation. All healthy animals showed values < 20 μg/mL.

#### Cow

Samples from 10 dairy cows (seven Holstein, two Montbellier and one crossbreed), mean age 4.9 ± 1.6 years-old, parity 3.4 ± 1.6, and BCS 3.4 ± 0.7, from a commercial dairy herd located in the southeast of Spain were used. All animals were at the last phase of gestation, apparently healthy and no lameness, mastitis, metritis, ketosis, or other health issues were observed. Blood and saliva samples were obtained 13 ± 7 days before calving (Before calving) and on the day of calving (At calving), between January and February of 2019, to avoid any change in the results due to seasonal reasons. Serum Hp and the total WBC measured by an automated hematological analyser (Advia 120, Siemens Healthcare GmbH, Erlangen, Germany) were used as indicators of inflammation. Healthy animals showed values lower than 20 mg/L and 11 × 10^9^/L for serum Hp and WBC count, respectively [50, 53].

### Statistical analysis

Data obtained from ADA measurements were analyzed for normality, giving a non-normal distribution. The changes due to the presence of EHNA at different concentrations were assessed by Friedman’s, followed by Dunn’s multiple comparison tests. ADA2 results obtained after manual and automated inhibition in the 33 samples (15 with low and 18 with high ADA2 activity) of porcine serum and saliva were compared by linear regression and Bland-Altman plot in which difference between methods was plotted against the average value. Unpaired Mann-Whitney test was used to compare tADA and isoenzymes results between healthy and diseased animals. The Wilcoxon signed-rank test was used to compare tADA and isoenzymes results between the two different measurements performed in cows. Spearman correlation coefficients (*r*) were calculated between ADA results and the biomarkers of inflammation. The correlations were considered according to the *r* value as very high (≥ 0.90), high (0.70–0.89), moderate (0.50–0.69), low (0.30–0.49), and negligible (< 0.30), following the Rule of Thumb [54]. Data analyses were performed using Excel 2000 (Microsoft Corporation, Redmond, WA, USA) and Graph Pad Software Inc. (GraphPad Prism, version 5 for Windows, Graph Pad Software Inc., San Diego, CA, USA). An α value of less than 0.05 was considered significant.

## Data Availability

The datasets used and/or analyzed during the current study are not publicly due to legal reasons but available from the corresponding authors on reasonable request.

## Abbreviations

ADA: Adenosine deaminase
tADA: Total adenosine deaminase
ADA1: Adenosine deaminase isoenzyme 1
ADA2: Adenosine deaminase isoenzyme 2
BCS: Body condition score
CRP: C-reactive protein
CV: Coefficient of variation
EHNA: Erythro-9-(2-hydroxy-3-nonyl) adenine
Hp: Haptoglobin
LLOD: Lower limit of detection
SAA: Serum amyloid A
SD: Standard deviation
WBC: White blood cell count
